# Portraying improvement in the management of chronic pain: A multi-modal longitudinal interpretative phenomenological analysis study

**DOI:** 10.3389/fpain.2022.901271

**Published:** 2022-09-20

**Authors:** Isabella E. Nizza, Jonathan A. Smith, Jamie A. Kirkham

**Affiliations:** ^1^Department of Psychological Sciences, Birkbeck University of London, London, United Kingdom; ^2^Kent Community Health NHS Trust, Ashford, United Kingdom

**Keywords:** chronic pain, interpretative phenomenological analysis (IPA), longitudinal, visual methodologies, participant drawings, pain management program (PMP)

## Abstract

Chronic pain is a common, profoundly disabling and complex condition whose effects on identity may explain the distress experienced by those affected by it. This paper concerns a study exploring how the relationship with pain and sense of self evolved following participation in a pain management program (PMP). Participants were interviewed at three timepoints: before attending a PMP, 1 month after the PMP and 6 months after the PMP. To facilitate a deep experiential description of pain and its effects, interviews were guided by participant-generated drawings of pain and Self. Interviews and drawings were analyzed longitudinally using interpretative phenomenological analysis. The evolving experience of participants was outlined through different trajectory types. Here we describe the upward and positive trajectory of three female participants who were able to regain control over their lives. From a state of psychological stress where pain was represented as an aggressive and oppressive presence, participants' drawings, their narratives and indeed their lives, changed for the best. Pain stopped being the main feature, they were able to integrate it into their lives, make important changes and find a new balance. The results demonstrate the idiosyncratic nature of chronic pain and offer a nuanced account of its links to the lifeworld of those living with it.

## Introduction

Pain is diagnosed as chronic when it persists for more than 3 months in the absence of progressive disease (such as cancer) or structural abnormalities (1). Chronic pain (CP) is a major health concern in most Western countries. An American National Health Interview Survey found prevalence amongst adults of CP and high impact CP (which limits life and work activity) to be 20.4 and 8% respectively (2), while in the UK severely disabling pain has been estimated to affect between 10.4 and 14.3% of the adult population (3). Both studies indicate higher prevalence for women compared to men, and older adults compared to younger ones.

Qualitative literature has generated much insight into the profound and far-reaching impacts of CP on the lives of those affected: people are dramatically and intimately changed by their illness and have great difficulties in coming to terms with having CP (4). To support those with CP, most English national health trusts offer Pain Management Programs (PMPs), multidisciplinary interventions aimed at helping people to learn to live with their pain. Evidence on PMP efficacy is however weak, mostly for the wide range of study designs and outcome measures (5). Interestingly, the impact of CP on identity that clearly emerges from qualitative literature (4) is not directly reflected in how interventions are conceived, delivered and evaluated. This creates the question of what the effect of attending a PMP might be in terms of the existential impact that CP has on the life of those living with it.

CP as a health condition is particular because it is invisible, difficult to describe, impossible to assess objectively and profoundly idiosyncratic in its effects. Visual methods, increasingly used in qualitative research (6, 7), are particularly suited to investigate CP, because they enable the unexplainable experience of pain to be examined and conveyed more fully. Drawings, in particular, offer a direct path to revealing feelings and emotions (8); like metaphors, drawings can act as a ‘safe bridge' to express painful feelings (9). Drawings of pain have revealed a complex imagery, with pain represented as an aggressive attacker (10) or an external malevolent object of torture (11), thus shedding additional light on the distressing descriptions of having CP provided through narratives alone. Such pain imagery vividly illustrates CP as an adversarial experience, characterized by a sense of impotence in which personal agency is lost to pain. In this study, drawings of pain were combined with drawings of Self to facilitate the expression of alternative narratives of participants' identities and personal worlds.

The medical definition of CP emphasizes its subjectivity and emotional impact (12), so a qualitative methodology, particularly a phenomenological and idiographic one such as Interpretative Phenomenological Analysis (IPA), is a very apt way of exploring the idiosyncratic aspects of the CP experience (13). If the aim is to understand change or the lack thereof, following an event such as PMP participation, a longitudinal design is the most appropriate (14). There is a small corpus of studies that have employed IPA longitudinally to explore changing CP experience after pharmacological treatment or an educational intervention (15, 16). Our study adopts a longitudinal design, to understand change after a PMP, by gathering data at three time points: before and after the PMP and 6 months after the PMP.

To summarize, this article presents results from a study where IPA interviews with drawings were analyzed longitudinally, to understand how pain and the sense of identity of participants with CP progressed after they participated in a PMP. The evolution of each participant's experience over the study's three time points was described as a trajectory, and three types of trajectories emerged from the overall study: an upward and positive trajectory, a negative and unchanging trajectory and a positive but complicated trajectory. This paper presents the detailed findings from the first upward and positive trajectory, by discussing the narratives and pain and Self drawings of three participants, Olga, Monica, and Jane, who showed a substantial and consistent improvement over time. The other trajectory groups are to be discussed in separate papers currently being prepared.

## Materials and methods

### Participants

Participants were 40–60-year-old women, unemployed and suffering from chronic pain for at least 2 years who had been referred to a National Health Service Community Chronic Pain Service (CCPS) in South-East England. Table 1 summarizes key details about participants. As you can see, although the data were gathered at equivalent timepoints, each participant arrived at the service with a long personal history of CP and the support they received was tailored to their needs. For instance, Olga attended tai-chi sessions and was referred relatively late to the PMP, while Monica received 1-to-1 psychological support.

**Table 1:** Details about participants in the upward and positive trajectory group.

**Pseudonym**	**Diagnosis**	**Age**	**Years with pain**	**Medication^a^**	**Non-medical treatments received from service^b^**	**Months between interviews 1 and 2**	**Months between interviews 1 and 3**
Olga	Fibromyalgia and seronegative rheumatoid arthritis (RA)	55	20	RA medication	tai-chi	6	11
Monica	Fibromyalgia	40	30+	Opioid painkiller, antidepressant, paracetamol and treatment for hiatus hernia	psychology	3	8
Jane	Fibromyalgia, degenerated disks, depression	47	3	Opioid painkiller, anticonvulsant, antidepressant, paracetamol and supplements		4	8

^a^The contents of the Medication column are for the most part derived from the participants' clinical records at the end of the study.

^b^All participants also had regular personal appointments with a clinical nurse specialist and participated in the PMP.

### Recruitment and data collection

Ethical approval for the study was granted by the NHS London-Stanmore Research Ethics Committee in 2015 (15/LO1872).

Participants were recruited during a pain education session that all people attend shortly after being referred to the service. Participants who expressed interest in the study during the session were given an information sheet, the opportunity to ask questions and were recontacted by the researcher a few days later to confirm participation. After a few months, and at the discretion of their care nurse, people can also be referred to a four-week-long non-residential PMP. Participants in this study were interviewed ~2 weeks after attending their education session, 1 month after the end of their PMP and then again 6 months after the end of their PMP. Interviews were held in surgeries and lasted on average 78 min.

At the start of the first interview, having signed the consent form and received reassurance that the artistic quality of drawings was not important, participants were left alone in a room for 15 minutes to create a drawing of their pain. They were given an A4 blank sheet of heavy paper and colored pencils, crayons and felt-tips, and were asked to “*draw a picture of what your pain feels like to you*”. The researcher then returned to the room and asked them to “*draw a picture of yourself as you are now*”, leaving them alone for 15 more minutes. When the drawings were complete, the semi-structured interview started. Focusing on one drawing at a time and starting with the pain drawing, participants were asked to describe their drawing, why it was drawn as it was and their thoughts looking at it; there were also questions on how the pain made them feel about themselves, how they thought others saw them and how they would have liked to feel.

When some participants became tearful, the interviewer gave them an opportunity to recover or the option to interrupt the interview, but all participants were happy to continue. At the end of the interview, there was also a debrief during which the researcher was able to verify how participants were feeling and answer any of their questions. No one expressed the need for further support.

The second and third interviews were similar, except that, in the second half, drawings from the earlier interviews were shown to encourage a reflection on change. All interviews were audio-recorded and transcribed verbatim. The interviewer (IEN) kept a reflexive journal and was clinically supervised by the third author (JK), a CP expert senior counseling psychologist.

### Analysis

All participants were assigned pseudonyms and, when necessary, the drawings were anonymized by electronic editing. For each participant there were three pain drawings, three Self drawings and three interview transcripts to be analyzed. Data were analyzed inductively, idiographically and longitudinally, before comparing cross-case.

The analysis of each interview started with an analysis of the pain and Self drawings using the framework for visual analysis inspired by compositional analysis proposed by Boden and Eatough (6, 17). Then the interview transcript was analyzed to make notes and identify personal experiential themes, as suggested by the IPA method, linking the themes to the drawings (14, 18). Here particular attention was placed on prospective views (considerations that emerged from reviewing newly created drawings), and, in later interviews, retrospective views (considerations that emerged when comparing previous drawings with new ones). Finally, for the longitudinal analysis of each participant, the tables of personal experiential themes from the three timepoints and the drawings were considered as a gestalt to identify the participant's individual trajectory in the study. At the very end, the different individual trajectories were compared cross-case and grouped into three trajectory types: an upward and positive trajectory, a negative and unchanging trajectory and a positive but complicated trajectory.

In the next section, results from the upward and positive trajectory group are presented case-by-case. Each case develops chronologically, so that what happened to a participant at the first timepoint is discussed before discussing the second and the third timepoints. Within each timepoint, the focus is first on the pain drawing and then on the Self drawing. All the drawings from each participant are also presented in a single table figure, which contains one row per time point, and a pain and a Self-column, to enable horizontal comparisons between the pain and Self drawings from a given time point and vertical comparisons between the pain and Self drawings from different time points. Comparisons between participants are developed fully in the discussion.

## Results

The upward and positive trajectory includes three participants who wholeheartedly embraced the change encouraged by the PMP and whose lives, in the following months, were transformed and normalized. Olga, Monica and Jane's stories and drawings share a consistent progress over time, although what constituted progress was different for each of them. As will become apparent reviewing their individual trajectories, they all started from a position in which the pain was represented as an aggressive and oppressive presence in their lives, and they appeared under great psychological stress in their initial Self drawings. Step-by-step, through attending the PMP, but not only, their drawings, their narratives and indeed their lives, improved. Pain stopped being the main feature of their lives, they were able to integrate it and find a new balance and sense of control. All women in this trajectory group embraced their time in the CCPS and their PMP attendance as a unique opportunity to make important life decisions, work on themselves and their situation and regain control over their life.

### Olga's journey: From despondency to planning her future

Olga stayed the longest time within the study and was the only participant to successfully wean herself off opioids after having taken them for over 5 years. She illustrated her journey with two sets of surprisingly simple stick figures that evolved from her having overwhelming pain and extremely low mood, to having less pain and a strong sense of purpose in her life.

#### Time 1

**Figure 1:**
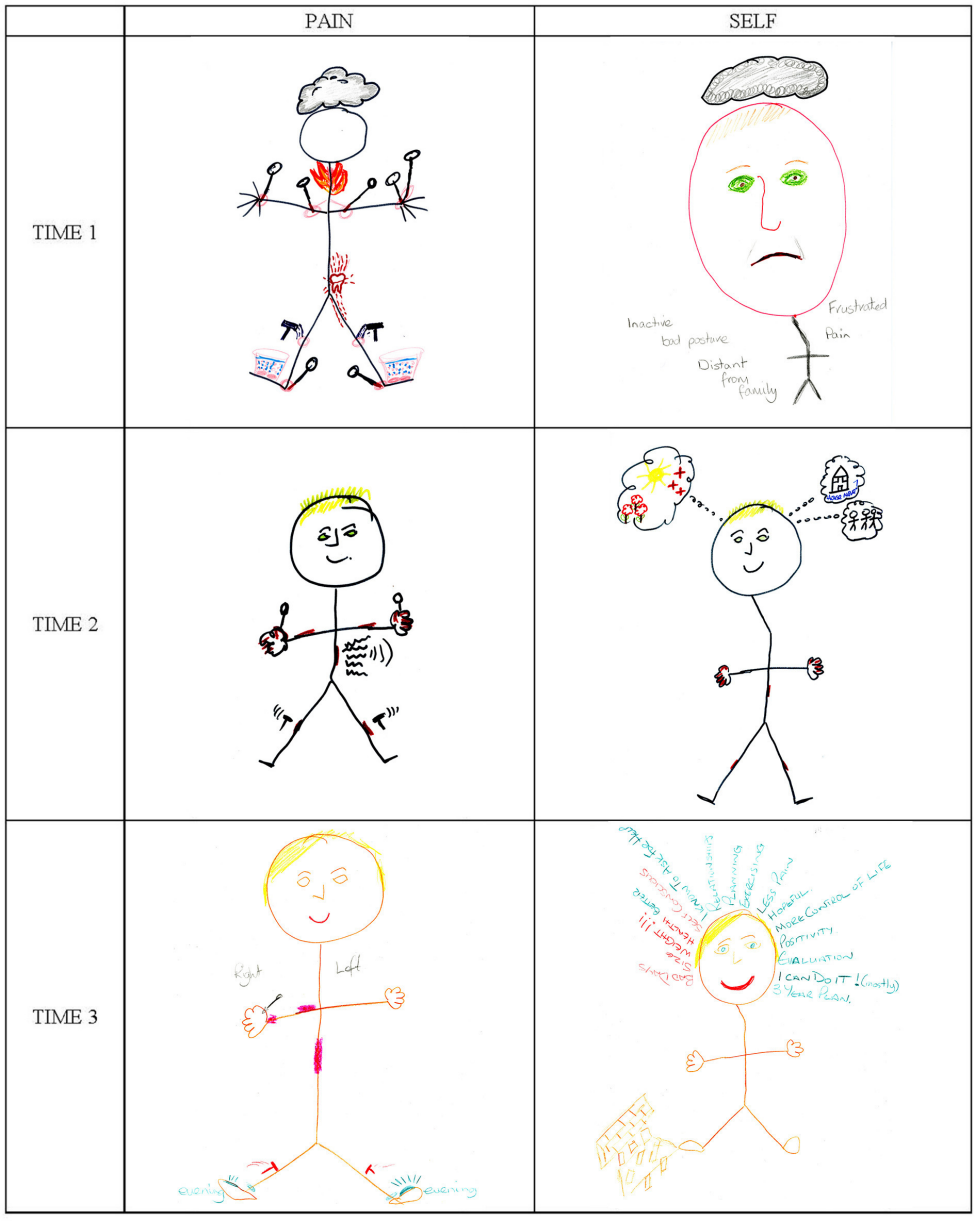
Olga's drawings of her pain and Self at Time 1 (T1), Time 2 (T2), and Time 3 (T3).

Olga's first pain drawing (Figure 1, Pain T1) is a faceless stick figure on which the pain is described via metaphors: pokers in her joints, hammers on her knees, flames on her neck, toothache at the base of her spine, buckets of fizzy water at her feet. The variety of symptoms and the violence of some of the metaphors (e.g., flames and pokers) suggest a very aggressive pain. Olga had carefully chosen what to draw:

I wanted it to be red, because it's angry and it hurts, and it's like somebody was pushing a hot poker or hot needles into my joints [Olga, T1]

Olga ascribed agency to the pain (“*it's angry and it hurts*”) and imagined “*somebody*” pushing hot pokers into her joints. Her pain was not only strong, it was separate from her. In fact, Olga as a living person does not appear at all in her first pain drawing, because the figure has no face and no identifying features. The drawing is literally just about the pain. Pain seemed to have taken over Olga's body completely, with things being done to her body by an external force.

Olga had been tearful during her first interview and profoundly upset by the effects that pain was having on her life. She described the cloud above the stick figure's head as a constant overhanging “*heaviness*” and felt the pain isolated her from other people. It was clear that her physical pain was compounded by a low mood, possibly a form of depression, a diagnosis that would fit well with the sense of emptiness expressed by the figure's blank face.

Olga's first Self drawing (Figure 1, Self T1), with its large head and small stick figure body, illustrates in more detail the impact of pain on her mood. The face has a forlorn expression, with a downturned mouth and Olga's green sad eyes. As with pain, a large gray cloud hovers above the head. This is how Olga described her feelings:

[tearful] I feel a bit angry […] because I think why have I got all of these problems, you know? I'd like it to improve, I would really like to think I could improve on it, I don't want this, sometimes it kind of makes you feel, if this is it, if this is the best you're gonna feel, I don't want it to continue, if this is it for the next 20 years, do you really want the next 20 years? [Olga, T1]

This quote is rich and captures the depth of Olga's despair and the sense of hopelessness that her “*problems*” were evoking. The problems are listed as labels on the drawing, they include pain, but not only. Olga was physically “*inactive*,” for her pain and because she was overweight. She disliked her body (“*I'm big, so I try not to think about it”)*, which is why it is out of proportion compared to the head and almost invisible, apart from having a “*stoop*” (the slight bend in the stick figure). Olga felt very self-conscious because her stoop caused a “*bad posture*.” Her physical limitations affected her ability to engage with her loved ones, making her feel isolated and “*distant from family*,” and her overall situation made her “*frustrated*”.

Going back to the quote, Olga felt that her current situation made for a life that was not worth living (“*if this is the best you're gonna feel, I don't want it to continue*”). However, her desire for something better (“*I would really like to think I could improve it*”) felt tentative, she wanted to change, or rather, she wanted to “*think*” that she could change but did not entirely believe that this was possible. Her impulse for change was weak. She referred to her situation using pronouns such as “*this*” and “*it*”, almost distancing herself from it, conveying a sense of impotence, as if her life were beyond her control.

At her first interview, Olga was suffering greatly both physically and psychologically: she was sad, in a body she disliked, feeling victimized by her pain and unable to react.

#### Time 2

By her second interview, which was 6 months after the first, Olga was feeling better. She had been referred to the PMP later than most and in the meantime her medication regime had been revised. Her second pain drawing (Figure 1, Pain T2) is, again, a stick figure with pain marks and, although it shares many similarities with the first (Figure 1, Pain T1), there are also important differences. Many metaphors are retained (notably, pokers and hammers), but some are omitted (e.g., no tooth and no flames), suggesting that the physical pain had diminished. Olga confirmed she was feeling better:

I think my pain has definitely improved to what it was when I was here last time, which again, that straight away increases the quality of your life [Olga, T2]

Olga saw a link between her pain levels and her mood. Her second pain drawing reflected her improved mood because the stick figure had blonde hair (like Olga), a smiling face and no cloud above its head. The absence of a cloud was also indicative of greater mental clarity from taking less morphine. The inclusion of a recognizable smiling face, where previously there had only been emptiness, suggests that the balance had changed between Olga and her pain: previously pain had been the protagonist of the drawing, while this was a picture of her with pain. It was as if she had reconquered some of the space previously occupied by the pain and was affirming herself in relation to it.

When asked how she explained the change, Olga said:

maybe coming off some of the pain killers, although you expect them to deal with all the pain but actually they don't always, do they? […] now I understand that they can cause as much pain as they relieve and, yes, and just maybe not having my brain quite so foggy with the drugs and stuff, […] when I did the PMP, it laid some of the fears for the future, so, ahm, I didn't realize how many different aids there were to help you with different things [Olga, T2]

Olga had experienced the detrimental effects of opioids, in terms both of mental fog and actual pain, a counter-intuitive effect of this type of medication difficult to believe. Participating in the PMP had also played a part, and, interestingly, the most important aspect for Olga had been learning about disability aids, such as kettle tippers. This detail speaks to the idiosyncratic nature of CP, whereby each sufferer is burdened by their own particular fears and concerns, but also illustrates how PMP participation can change participants' perspective about their future, as we shall see when discussing Olga's second Self drawing below.

Olga's second Self drawing (Figure 1, Self T2) is, again, a stick figure, but this time fairly proportionate and with hands and feet. The figure has Olga's blonde hair, green eyes and a happy smile. Above the head there is no dark cloud, but three thought bubbles which, from left to right, contain a shining sun with flowers and red crosses, a house with the text “*house move?*” and three other figures identified as Olga's family.

Although simple, Olga's drawings were always well thought-out. As previously, her Self drawing gives us insight into what was on her mind. Of the first bubble she said:

this is my future, I feel like it's a little bit more rosy, I know that there's a little bit of help out there should I need it, and obviously the sun is shining [Olga, T2]

The bubble included roses because her future looked rosier to her; the red crosses (meaning first aid), were the “*little bit of help out there*” from the CCPS and the disability aids mentioned earlier; and the sun was shining because she felt hopeful about the future.

The second and third bubbles illustrate issues unrelated to health that Olga discussed during her interview: whether to relocate and other family matters. What was notable here was Olga's focus beyond the boundaries of her own bodily reality, her new-found closeness with family and her future-orientation.

The stick figure's body in Olga's second Self drawing (Figure 1, Self T2) has its hallmark stoop and is scattered with pain marks. Olga said that the pain marks showed how well she was feeling, despite the pain. She did not discuss the stoop but, for the first time, talked of possibly losing weight:

I've got the motivation, because I know the quality of my life will improve, but I just can't, or I haven't at the moment clicked it the right place here [head] [Olga, T2]

Olga, who would be classed as obese, knew that her weight had an impact on the pain and on her overall quality of life, yet during her first interview she had only touched on the topic. This time she discussed needing a structured approach to dieting, but, despite feeling motivated, she was not psychologically ready to address the problem. There was a sense of purpose and self-awareness in Olga's words that seemed promising and boded well for the months to come.

Overall, at her second interview, Olga was feeling visibly better in herself. Her pain had reduced, and her mood had improved, she was smiling in both drawings, enjoying life and looking ahead.

#### Time 3

At the third interview 5 months later, Olga's situation was even better. Her third pain drawing (Figure 1, Pain T3) is quite like her previous one, but lighter, because drawn in pencil rather than felt-tip. The lightness of the drawing, especially the pain marks, was deliberate to show how the pain had changed:

I definitely wanted that in pink, because I wanted it to be a lighter color than it was, not in red, because the red I feel is the real angry color [Olga, T3]

Since Olga's pain had changed in intensity, it required the use of milder colors; it had also changed in aggressiveness, so the red was no longer of the “*angry*” type but a milder pink. The figure, clearly identifiable as Olga, appeared larger, with a smile and lighter compared to previous ones, because instead of a black felt tip, she had used a softer skin-colored pencil mark to draw the outline, which gave the figure a “natural” look. This normalization reflected a change in Olga's narrative: her focus was no longer the pain but her weight-loss plans.

Looking across Olga's three pain drawings (Figure 1, Pain) it is interesting how they all present the same subject, and the subtle changes in tone and detail define a positive trajectory, over which the pain, from being a strong, depressing and depersonalizing presence, had reduced, allowing Olga to gently re-emerge and take center stage with a large smile, ready to live the rest of her life.

Olga's final Self drawing (Figure 1, Self T3) provides more detail on her plans. As always, it is a stick figure, this time drawn in brown and with a wide smile. Compared to previous Self drawings (Figure 1, Self T1 & T2), the figure has full hands and feet, the characteristic stoop is less accentuated, and it has no pain marks. To the left of the figure's foot there is a rough representation of a wall, with some bricks lying around. Above the head there is a halo of words. The words in red are negative things (“*bad day”*, “*size*”, “*weight!!!”*, “*health*” and “*self-conscious*”), while the green words are positive things (“*better (for health)”, “I know to ask for help”*, etc.).

At her third interview, Olga was smiling like the figure in the drawing, feeling in control of her life and hopeful. She had successfully completely weaned herself off opioids and, with her husband, had devised a “*3-year plan*” to improve their wellbeing, which included losing weight. Olga was cautiously optimistic about succeeding in her intent, so she added “*(mostly)*” to the “*I can do it!*” statement in the drawing. Previously her motivation to lose weight had been lagging because she had not “*clicked in the right place*,” now she felt ready to tackle her weight loss project:

I know I've gone on about my weight every time I've seen you but I really feel like I've got my head around it now, yes, I'm going to be able to make a difference to myself [Olga, T3]

Weight loss had hardly been prevalent in Olga's previous interviews, but in the last months it had probably been central to her thoughts and now it was her focus. In her drawing, three of the five red negative words in the figure's halo are weight-related (“*size*”, “*weight*” and “*self-conscious*”).

A notable (and new) aspect of her third Self drawing was the contrast between positive and negative elements. It was as if Olga had engaged in a battle with herself: the red words in her drawing were her opponents and those in green were her allies. Since there were more of the latter, she was optimistic about being successful.

The meaning of the crumbling wall in Olga's final Self drawing was more ambiguous. Initially she described it as “*the thing that stops me from doing things, the negativity in my life*”. When comparing her new Self drawing to her previous ones, she explained the wall as being the diminished pain, explaining that she was “*not focusing on it as much”* and “*focusing on other things as well*”, whereas before she had been “*completely enclosed*” by her pain. Now that she was no longer taking morphine and had less pain, the wall had crumbled, she was able to focus away from her pain, to “*hop over*” the wall and get on with her life. This was a pivotal moment:

I am much better that than I was, I feel better in myself, my pain has improved, I feel like my life is sort of at a bit of a turning point really, I'm in control of what I do and what's happening to me, I can make it change [Olga, T3]

#### Summary

Olga's closing tone in this last quote feels miles away from the despair and impotency of her first interview. She was not only better, she felt in control and empowered to bring about the changes she needed in her life. Her overall trajectory, so powerfully illustrated in her simple pain and Self drawings, had been extremely positive. The pain that initially was controlling her life was dramatically reduced, her mood lifted and gradually she was able to re-engage with her life and embark on making important changes that would further improve her quality of life.

### Monica's journey: From resistance to self-compassion

Monica's time in the study was a transformative journey of self-discovery. Her pain drawings evolved from pain being an aggressor to resist, to pain being a manageable challenge. In parallel, her Self drawings evolved from a precarious balance to a symbolic revelation of Monica's identity.

#### Time 1

**Figure 2:**
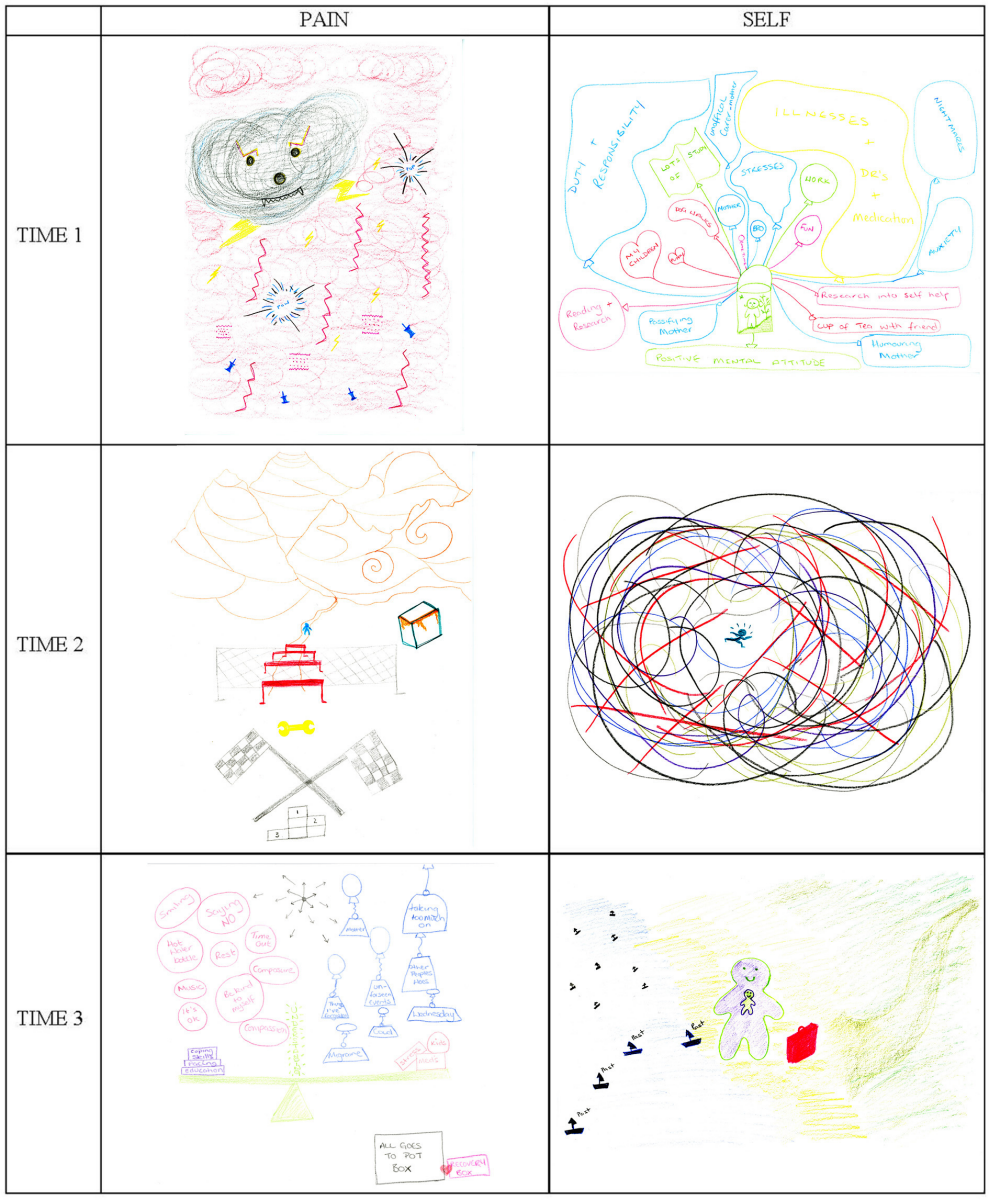
Monica's drawings of her pain and Self at Time 1 (T1), Time 2 (T2), and Time 3 (T3).

Monica's first pain drawing is dense and slightly eerie (Figure 2, Pain T1). On the left it contains a gray cloud with a teddy bear-like face, and prominent vampire teeth; the rest is a dense pink fog interspersed with shapes: blue pins, yellow lightning, pink zigzag lines, small “*pop*” and “*pow*” explosions. There is a chilling contrast between the comic-book tone of the drawing and Monica's description of it:

some of the pain can feel cold and some of it can feel aggressive […] it's like an aggressive being just sitting there waiting to… “What shall I do with you today?” [Monica, T1]

Along with the physical aspects of her pain (e.g., “*cold*”), Monica described the ‘teddy bear' as aggressive, spiteful, agentic, and rather malevolent, intent on spoiling her life daily (“*What shall I do with you today?*”). Although some elements of the drawing seem playful, it conveys the sense of a pain which is widespread, complex, and aggressive.

During her first interview, it became apparent that Monica was not coping well. She was a single mother of two young children, caring for her mother, studying and working as a volunteer. With so many things going on, she was finding it difficult to focus on herself and her pain. Her first Self drawing (Figure 2, Self T1) is a dense and elaborate diagram of all the things she was juggling. Monica represented herself as a minute figure in a green basket attached to many balloons occupying the whole page above and beside her. The balloons are color-coded, with negative ones in blue (i.e., “*duty and responsibility*”, “*stresses*”, “*mother*”, “*brother*”, etc.) and positive ones in red (i.e., “*reading and research”*, “*children*”, “*puppy*,” etc.). Other important elements such as “*study*” and “*work*” are in green, while the largest balloon (“*illnesses* + *dr's* + *medication*”) is yellow. The balloons appear constrained, oppressive for how they fill the space around the small figure and give the image a claustrophobic quality. Also, the blue and yellow balloons occupy in proportion considerably more space than the others, particularly the red ones which represent love and self-care.

The “*duty and responsibility”* balloon on the left is large because Monica spent most of her time taking care of her family (“*they're all my responsibility because they're hopeless*”). Four of the negative balloons concern her mother (“*mother*,” “*unofficial carer-mother*,” “*pacifying mother*” and “*humoring mother*”), with whom Monica had a strained relationship. Monica spent little time looking after herself: in the drawing, her red “*me time*” balloon is so small that the label is written on the string. Having given herself entirely to others, Monica felt that there was none of her available to look after herself: “*I kind of need me more than anybody else does and I don't have time for me!”*

Could a simple lack of time explain why Monica wasn't taking care of herself? This quote offers insight on what was occurring at a deeper level:

I should be allowed to go and be sick and get over it [laughs] in an ideal world. But I don't want to be like other people, I don't want to give in to it, because if I give in to it, I've lost my self, because if I do that, there won't be me, there will just be the illnesses and the pain and the yuck. […] That balances out quite nicely, without me even realizing it [Monica, T1]

This quote captures the existential battle that Monica was fighting against her illness. She perceived her illness and her Self as two separate mutually exclusive entities. She knew that she should look after herself, yet she felt unable to do so because she was afraid that the illness would take over her life and that she as a person would cease to exist (“*if I give in to it, I've lost my self* ”). Despite her “*ideal world*” claims, to stop her illness from winning the battle, Monica was not allowing herself to invest time and energy into caring for herself. Hers was an act of resistance.

Monica's drawing of her Self made perfect sense in these terms. Pain (yellow balloon) occupied a large amount of space in the picture, but it was almost invisible. She explained that she had drawn the illness and medication balloon in yellow because it was a color she “*really detested*”, but, at the same time, it was a color that allowed her to pretend the balloon did not exist. She knew that the apparent “*empty space*” of the yellow balloon was just an illusion and that it could not really be filled by anything else. What she could do was fill the rest of the space with other things, so that the yellow balloon, however big, could not expand and occupy more space. Filling her day to the brim was Monica's way of keeping her illness at bay and resisting it.

It felt as if Monica were under constant threat of being annihilated by her illness. From the outside, her life appeared too full, but from Monica's perspective, all the elements were well balanced (“*that balances out quite nicely*”). Monica described her system of balloons as “*compartmentalization*” which she considered a successful strategy to keep a good psychological balance. The miniature idyllic environment surrounding the green figure at the bottom of the drawing was a symbol of her “*positive mental attitude*,” also part of her psychological survival toolkit.

At the start of the study, Monica was desperately resisting the attacks of pain toward her body and her very being. She was defending herself by filling her life so compactly that a psychological balance of sorts was achieved, although seen from the outside it appeared precarious and unsustainable.

#### Time 2

After attending the PMP, Monica was referred for individual psychological support within the service, where she had the opportunity to explore what was happening to her. Her second pain drawing (Figure 2, Pain T2) shows the results of her efforts to manage her pain. Pain is presented as an obstacle course, where every day is a race toward the finishing line:

then you get down to here […] and then you get all these hurdles put in front of you and so, “okay, right, okay, we'll get over some of these hurdles,” […] and then something happens and there's a spanner thrown in the works [Monica, T2]

The second-person voice in this quote suggests detachment and the expression “*you get all these hurdles put in front of you*” is passive, implying that Monica was still seeing the pain as an external entity exercising control over her.

Monica was still learning. Although her situation was far from ideal, she was dealing with the pain by staying focused on the present, confident that eventually she would be able to get down from the mountain and finally have a ‘good day,' reaching the podium in her pain drawing.

Monica's second drawing of Self (Figure 2, Self T2) is the epitome of disruption, especially compared to her pain drawing (Figure 2, Pain T2) and her previous Self drawing (Figure 2, Self T1). It is a bundle of lines surrounding a tiny green figure on the run. The image is strong, colorful, and scary, but also playful in how the figure resembles Keith Haring's dynamic figurines. The day of her second interview, Monica was in a crisis because she was organizing her mother's relocation. She described the bundle as a “*hornet's nest*” and “*being in the middle of a tornado*.” Despite the catastrophic metaphors, Monica was confident that the situation would soon be resolved.

The PMP and counseling had improved Monica's ability to cope. Looking at her second Self drawing (Figure 2, Self T2), she said:

I would have panicked about something like this before and I wouldn't have drawn it, because it shows that “Oh my God, you're insane!” No, I am not, what is going on here is that there is so much going on that this is the only way I can express it to you, but actually I am all right, I am not upset about it, I am not climbing the walls, this is just how it is […] it [PMP] has given me an allowance to accept that that's okay [Monica, T2]

Monica had gained the understanding that not having everything under control could be normal and acceptable. She was not necessarily coping better with the chaos; she was interpreting her anxious response to chaos as within the range of “normality” and not as a sign of deteriorating mental health. By saying that previously she would not have allowed herself to create such a drawing, Monica was also shedding new light on her previous representation of her Self (Figure 2, Self T1). This second Self drawing (Figure 2, Self T2) felt much less constrained and disturbing than the first, the irony and freedom of the wild lines seemed energetic, healthy and refreshing in comparison to the oppressive concentration of balloons. Monica confirmed that despite being under pressure, she felt relaxed and accepting of her feelings. Listening to herself had been one of her main learning points from the PMP:

It's listening to me and how I am actually feeling and not ignoring it, whereas before I have always ignored it and just carried on, but I am learning to listen [Monica, T2]

Previously too busy looking after others and resisting the pain, Monica was starting to “*listen”* to her needs and, occasionally, when her body would tell her to, she would stop. She had also started to change how she interacted with her mother, by saying no to some of her requests.

Although the pain still had a strong and limiting presence in Monica's life, her pain drawing reflected how she was learning to live with it. Her Self drawing showed the confusion of a challenging moment, but also her new-found ability to cope with chaos.

#### Time 3

Monica's third pain drawing (Figure 2, Pain T3) represents her new pain management equilibrium. It includes a large scale, with various elements balanced on either side: “*stress*”, “*meds*” and “*kids*” on the right, the three constants in her life, kept into balance by “*coping skills*”, “*pacing*” and “*education*”, new skills from the PMP. Hovering on the right there are ‘negative' blue weights hanging from balloons (e.g., her mother), to be balanced, on the left, with ‘positive' pink bubbles of behaviors and attitudes, such as “*composure*” and “*compassion*”. It is interesting to note how the negative weights on the right are external forces over which Monica had no control, while the positive bubbles on the left are resources through which Monica could exercise agency. Superficially, this drawing resembles Monica's first Self drawing (Figure 2, Self T1), with its numerous balloons. Yet here the components are tidier and less oppressive; there is plenty of white ‘breathing' space around the scale and the sensation looking at it is indeed of balance.

Here is a practical example of how the balance was working for Monica:

I did not sleep at all last night, I couldn't get comfortable, my brain wouldn't switch off, I wanted to take my legs off, so rather than doing what I'd normally do and get screwed up about it, I was listening to my relaxation music. It didn't work, I didn't sleep, but it did work because I wasn't stressed out, I was nice and relaxed. […] Normally I'd have woken up […] irritated, and strung out, and really tired, and really grotty and… and I'm not, and I've got a really busy day at work today, but I'm quite happy […] So just a little bit of learning, a little bit of understanding and being nice to myself [laughs] has made it something to be dealt with, rather than something to fight against [Monica, T3]

This quote really brings the concept of self-management to life: faced with a difficult night of pain and restlessness, Monica turned to her tools (“*my relaxation music*”); although the tools did not eliminate her symptoms (“*it didn't work, I didn't sleep*”), her emotional response to the symptoms changed (“*but it did work because I wasn't stressed out*”); as a consequence, the downward spiral of increasing distress that a bad night could have triggered (“*irritated and strung out*”) was not initiated, and in the morning Monica felt able to face her day with optimism (“*I am quite happy*”). With the tools and the “*understanding*” acquired through the CCPS and PMP, Monica had found a new way of living with her pain and complex life that felt balanced. Monica's relationship with her pain had changed substantially: pain had become “*something to be dealt with, rather than something to fight against*.”

Self-compassion (“*being* nice *to myself* ”) was a key component of Monica's ability to deal with the uncertainty of CP. The “*all goes to pot box*” on the bottom right of her third pain drawing (Figure 2, Pain T3) symbolizes Monica's awareness that, however well-balanced her new situation, there was always a risk of something disruptive occurring. For such cases, she had her pink “*recovery box*,” the ability to accept that not everything can always go to plan. When something went wrong in her day, Monica had learnt to stop and, most importantly, be “*very nice*” to herself.

Self-compassion was a new skill for Monica and her third drawing of Self (Figure 2, Self T3) sheds light on the underlying psychological transformation it entailed. At the center there are two figures, one inside the other like a Russian doll, standing next to a red suitcase. On the left there is a blue sea with small black boats, labeled “past” and on the right there is a path winding through pale green land. Monica explained that the two figures represented her: the outer figure was the person she presented to the world, while the inner figure was her “*Little Me*”, her true Self:

this is what carries me, really, I seem to exist inside me, I always have done, what's more important is how I feel inside as opposed to the person I wear […] my Little Me gets hurt a lot, a lot of my life I disassociate how I feel from who I am, I have done it for a very, very long time. I've had a horrible little life. So I'm in there and this bit is what carries my Little Me, because the outside bit gets battered and bruised and abused and that's the bit that hurts […] the little bit, it's the bit that gets protected inside and the little bit is happy at the moment [Monica, T3]

This is a complex quote, reflecting the understanding that Monica was developing during therapy. Her traumatic past, the extent of which had not been apparent up to that moment, became central to her representation and discussion of Self. Earlier abusive relationships had caused her to protect her most vulnerable Self from the world by enclosing it in a carapace-like external Self visible to others. Most of the time she had been playing a role for others, trying to live up to their standards and pushing herself beyond her own limits. Being “*nice*” to herself was part of the process of embracing her “*Little Me*” and giving it the love and care that it had never received before.

As part of her inner journey, Monica had reassessed the people in her life and cut many old toxic relationships (“*because of how they were, who they were, and how they made me feel*”), symbolized by the little black boats departing by sea in her Self drawing (Figure 2, Self T3). The red suitcase contained what was worth keeping. As part of her self-analysis, Monica had accepted that even negative experiences had taught her something and added to who she was today.

Monica's third interview felt like a breakthrough: she was making peace with a difficult past, that she had briefly mentioned but never represented in her Self images. Her early Self drawings had reflected her efforts to keep her complex emotional world in balance (Figure 2, Self T1) and, later, accepting that control was not always possible (Figure 2, Self T2). Her third drawing was, literally, a drawing of her Self (Figure 2, T3): with her fragilities, her difficult past, but also with a clear path into the future. It was a serene and spacious drawing that was promising. When reviewing the three drawings together, talking about the “*duty and responsibility*” balloon from her fist Self drawing (Figure 2, Self T1), Monica recognized that she had a choice (“*nobody's holding a gun to my head and making me do it, I have a choice in this*”). This view of responsibility as a choice was in sharp contrast with how Monica had described the support she gave her family, particularly her mother, during her first interview (“*they're all my responsibility because they're hopeless”*). In her second interview, she had described learning to say 'no' to her mother, now she felt empowered and recognized her agency in shaping her own life, including her family relationships.

#### Summary

Over the course of the study, from an external aggressive being, pain had been transformed into an integrated and manageable part of Monica's life. In parallel, Monica had embarked on a journey of self-discovery which brought her face to face with her own fragilities and from which she was emerging as an assertive woman determined to take care of herself.

### Jane's journey: From dark oppression to serene normality^1^

Jane's journey was one of the most successful: from a very low and lonely place, in which pain was an oppressing presence, she was able to regain an overall sense of control and naturalness. Her pain drawings evolved to reflect the increasingly less important presence of pain in her life while, in parallel, how she depicted her life in her Self drawings changed from a dark arduous path to a lighter more natural one. Note that a more extensive analysis of Jane's pain trajectory, has already been published elsewhere (19).

#### Time 1

**Figure 3:**
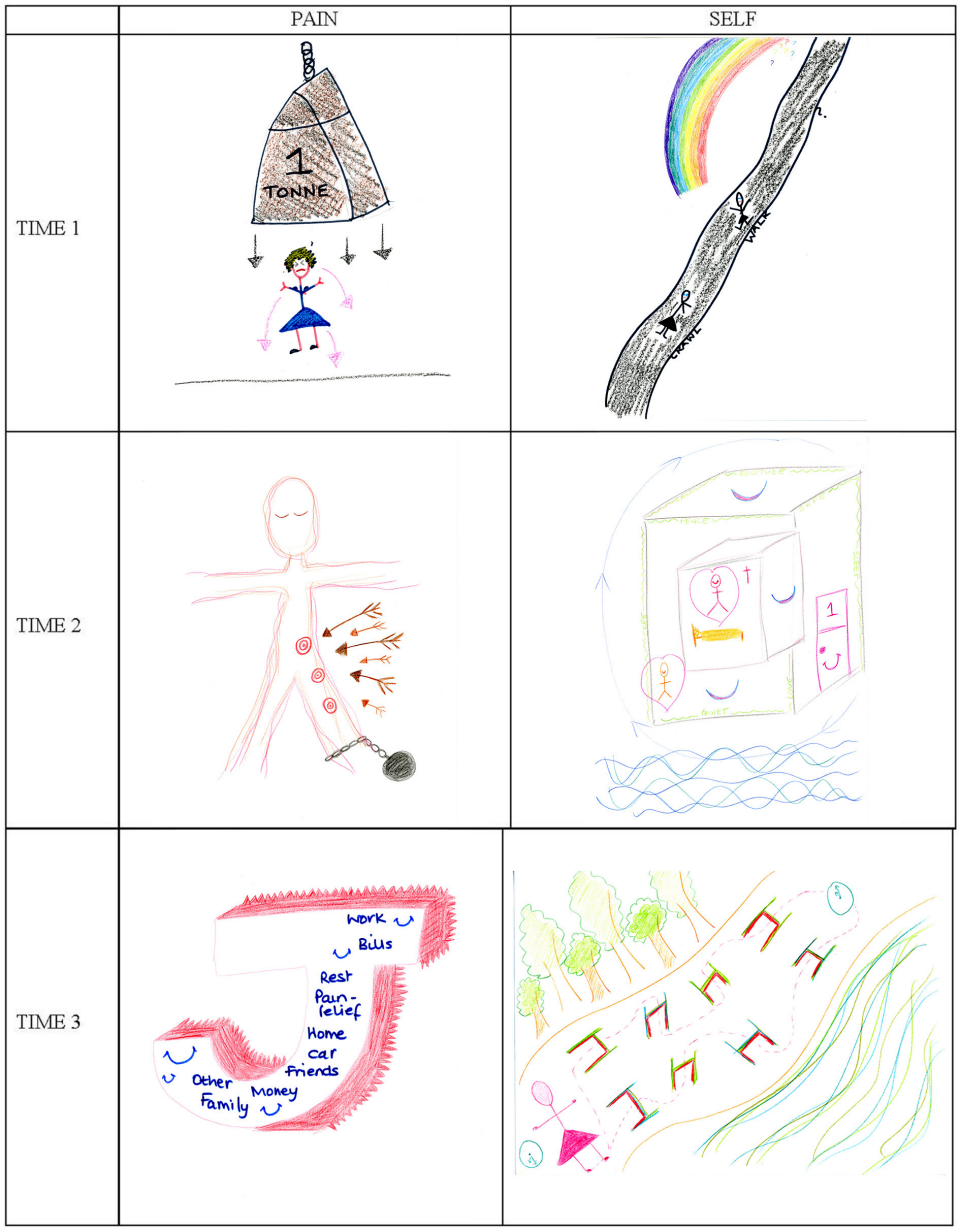
Jane's drawings of her pain and Self at Time 1 (T1), Time 2 (T2), and Time 3 (T3). Jane's pain drawings have already appeared in Nizza et al. (19) and are published with permission from Sage Publishing (c).

At the first timepoint, Jane drew her pain as a heavy one-ton weight hanging above the head of a small stick figure (Figure 3, Pain T1). The weight was disproportionate in comparison to the figure and the arrows suggest a downward movement, as if it might crush her, while she resists with flexed muscles. Jane described her pain as “*confining*” and “*crushing heavy*.” She equated the arrows projecting from the weight toward and around the stick figure's body to a cloak:

Then when you're kind of cloaked in this sort of pain, you haven't got the energy […] you're so focused on this [weight] […] you're trapped in this sort of bubble of what you want to do, what you can do and what you actually feel like doing [Jane, T1]

The pain was enveloping Jane and keeping her trapped, attracting all her attention, forcing her look inwards, limiting her energy. There was a discrepancy between what she wanted to do, could do, and actually felt like doing, pointing to an effect of the pain on Jane's mood.

The sense of feeling overwhelmed is echoed in Jane's first Self drawing (Figure 3, Self T1). It includes two stick-figure versions of her positioned on a long dark path that develops uphill across the page: the first figure is crawling, the second is walking, and further along the path there is an interrogation mark, questioning what lies ahead. A half rainbow hanging over the path ends somewhere beyond the page, as if hope had been interrupted:

when you've got this pain all the time [pause] it's hard to be sort of optimistic […] there are times when quite honestly I don't even want to know what is at the end of there [path], I'm not, I said to my GP actually, I want to see my son grow up, I want see him get married, have a career, I want to see him do all these things, but quite honestly, if I could fast forward through it all to the end, I would […] just get to the end and just let me have a rest, just stop, enough [Jane, T1]

Jane's life with pain felt like a dark and difficult path, where she was finding it hard to look ahead and be hopeful. She had lost her desire to savor even her young son's major milestones, so she was prepared to zoom through them to reach “*the end*” and finally have some respite. Although she clarified that she was not suicidal, Jane felt the need to “*rest*” because living was a struggle for her.

Jane commented on the sense of loneliness in her Self drawing: “*there's no background to it, there is no [pause] no trees, no pictures of anybody else, just me, pain and an uncertain future.”* Her sense of isolation was exacerbated by living with a partner who did not “*really understand”* her plight and feeling unable to effectively assert her own needs with him.

At the start of the study Jane was crushed, overwhelmed, and isolated by a pain that was preventing any form of “*normal”* life. She felt trapped in a dead-end situation: with a crippling tiredness, an unsatisfactory relationship, and an uncertain sense of her future.

#### Time 2

At the second timepoint, Jane drew her pain as an ethereal Christ-like figure with a ball and chain tied to its left ankle and some targets down its left leg (Figure 3, Pain T2). The impact of the pain appeared more limited compared to earlier: she said that the pain was “*direct in certain places*” and from there “*blurring everything else*,” suggesting that, although still present, the pain was now localized and less overwhelming (e.g., no longer “*crushing*,” but “*blurring*”). It was as if the weight from her first pain drawing (Figure 3, Pain T1) had lifted from her head and was now simply tying her down.

She also described the body of the figure in her second pain drawing as “*bisected*”: half linked to her painful past (with the pain marks and ball and chain), the other free half being her positive “*going forward side*”:

I've only done that on one side because of how my life is changed over the past few weeks […] this half of my body is more positive […] and that's the sort of going forward side [Jane, T2]

By her second interview, Jane's life had changed radically. During the PMP she had bonded with other CP sufferers, and this had given her the strength to leave her partner and move out of his home. She had also been offered a part-time job by a relative, which had the effect of “*reinforcing the positive side*” by making her feel supported by others.

When comparing her first and second pain drawings (Figure 3, Pain T1 & T2), Jane said, “*I've isolated the pain*,” and suggested that before she had “*allowed the pain to take over*.” In these expressions there is a marked shift in agency from the pain to Jane: where previously the control had been with the enveloping cloak of pain, now that the pain was more localized, the space it occupied seemed to have been reduced. Jane ascribed this shift to the PMP having provided her with tools through which she had been able to “*take ownership*” of the pain and “*put it in its place*”. The result was a more balanced view of herself, reflected in the symmetrical posture of the figure in her second pain drawing. Although still in pain with half her body heavy from the ball and chain, this was counter-balanced by new resources, which allowed Jane to self-manage her pain and feel more empowered.

Breaking up from her partner had been a release for Jane. Her second drawing of her Self (Figure 3, Self T2) illustrates her new living situation: by the sea, in her own flat (outer cube), with her beloved son and in her own bedroom (inner cube):

I just shut that door, that's my room, that's my space. [..] I am quite happy in my single bed and it's really lovely in there […] I've got solitude, safe, sleep, love, quiet and care and calm, because they're the things that I want in life, they're the things that […] I haven't had for quite a long time […] I've kind of got to the end of my rainbow [Jane, T2]

The words listed by Jane in the quote appear along the perimeter of the external cube. Her home and bedroom were places where she could experience a tranquility and a security that had not been possible while she was living with her partner. By deciding to break up and live on her own, Jane had finally affirmed her own needs over the needs of others. This impetus had extended to her relationship with her son, who appeared in her Self drawing in a heart but outside the inner cube, because new understandings acquired during the PMP had redefined how she conceived her role as a mother:

…it's okay to have, say, a bad day, it's okay to say I am going to bed, you know, tea is not happening tonight […] I don't have to worry about, you know, well I am the mum here, I should be doing x, y, z. [Jane, T2]

It was as if at the PMP Jane had been given permission to behave and think of herself differently. Her previous beliefs about what her mother/partner role entailed had led to behaviors that were damaging Jane physically, for instance by forcing her, when having a “*bad day*”, to stay up rather than go to bed. She took responsibility for her previous behaviors (“*I* was *making life harder for myself* ”) and was now recognizing her physical needs and acting accordingly.

The Self in Jane's second picture was a new version of her: free from her previous role constrictions, focused on her own needs and happy to the point of ecstasy. She described her new home as “*her sanctuary*” and dotted it with smiles and hearts. As Jane pointed out, the drawing contained the colors of the rainbow of which she had previously questioned the existence. The end of the rainbow had been reached. As the arrows around the cube in her second Self drawing showed, she had come “*full circle*,” finally coming home, both physically and metaphorically.

Jane's drawings of pain and Self at the second timepoint convey a new serenity and sense of control over her life. The role of pain was more contained than previously, and her Self drawing pointed to a newfound calmness and self-focus. The PMP had stimulated her to make substantial changes and, more importantly, to achieve a new positive outlook.

#### Time 3

Five months later, at the third timepoint, Jane's life appeared to have stabilized. She drew her pain as a three-dimensional letter J, her initial, with its depth colored in red and spiky teeth in the background (Figure 3, Pain T3). By representing herself (the letter J) as a working front, with all the key words of a ‘quasi-normal' life, Jane had been able to relegate the pain to the background:

People can't see it, which is why I've done it behind me, it's there and it's sharp, and it is all over, but people, they don't see it, because I don't whinge and I don't moan about it [Jane, T3]

Jane felt able to live her life without making others aware of her pain, which she considered an achievement. The words she added to the front of the letter J provide insight into what was important to her at the time: there were names of family members (which have been blanked out in Figure 3), because the improvement of her pain and general wellbeing had been accompanied by a re-kindling of her family ties; there were words such as “*money*”, “*car*”, “*work*” and “*bills*” to emphasize a return to normality; but there were also “*rest*” and “*pain relief* ” to indicate that, although not visible to others, pain was still an ongoing concern of hers.

Over the course of the study, Jane's pain had not ceased, but had been transformed from being an overwhelming presence, to being a part of her life that she felt control over and could conceal from others, within a routine of quasi-normality.

This new sense of normality also emerges from Jane's third Self drawing, which is a soft-colored representation of her “*path through life*” (Figure 3, Self T3). The drawing depicts a slightly uphill white path, with colorful obstacles, skirted on the left by a wood and on the right by the sea. A pink stick figure representing Jane stands at one end of the path, next to a clock. A dotted line weaves its way from her, through the obstacles, to another clock at the end of the path and back.

I put the trees and the sea because they're ongoing, they never change and they keep going and going, so this is like the path through life and the two clocks are the beginning of the day and the end of the day, and these are hurdles, because whichever way I turn, every day, it's just hurdles, they're not insurmountable hurdles, they're hurdles nonetheless [Jane, T3]

Jane was describing her daily routine, weaving her way over hurdles, in an unchanging pleasant environment. Although this description may seem low-key, particularly compared to her blissfulness at T2, a regular alternation of small and large obstacles, within pleasant surroundings well-exemplifies the serenity that often underlies “normality”. The obstacles Jane was facing were the ones she had listed in her third pain drawing (Figure 3, Pain T3): having to work, pay bills, manage a home, money, and so on. Considering that Jane had started her journey in the study on a dark path, to find her just 8 months later in control of her pain and working again almost full-time was astounding.

From being unemployed and financially dependent, Jane had become employed and running her own home. In her time off, she was also providing care for her sister's toddler:

I can't say no, because there's nobody else she can ask […] that was originally my day to just stop and do nothing [Jane, T3]

Was Jane's sense of sisterly obligation pushing her back into old behavior patterns, after having realized the importance of prioritizing her own needs at T2? Relationships of mutual support are fundamental building blocks of a “normal” life, so Jane's choice of helping her sister, despite adding to her hurdles, was another sign of regained naturalness and normality. What had changed was how Jane felt in control of her duties. When reviewing her third Self drawing alongside her previous ones (Figure 3, Self), she observed:

there's no black on this picture at all, my road isn't black, they're all natural colors, that's life, isn't it? Green and blue. So that's, that's the way of life, that's natural, I know what I'm doing, I'm just stepping over my hurdles, not crawling on a black road anymore [Jane, T3]

The black path leading off the page to emptiness from T1 had been replaced at T3 by a “*natural*” white path. Her mood and outlook at T3 were normalized, and, more importantly, she was expressing a sense of control over her life (“*I know what I am doing*”): she was “*just*” stepping over her hurdles and owning her normality. The inner renegotiation of her roles, that had led to changes at T2, had been metabolized and she felt once again able to take care of others without damaging herself. It felt as if her previous caring Self was cautiously starting to emerge again.

#### Summary

Jane's pain and Self drawings show a very positive trajectory. At the start of the study her pain had been overwhelming and she had felt dispirited, with no sense of future. The PMP stimulated her to make important changes to her life as a result of which at T2 her pain appeared more contained, and she had acquired a home of her own and a new outlook that made her feel elated. By her third interview, she had settled down, was able to handle her pain in the background and was in control of her life again, facing the obstacles of “normality”. There had been a substantial shift in power during the study: where previously the pain had controlled Jane's being, by the end she was able to assert herself over the pain and feel in control of herself and her life.

## Discussion

There are many parallels between the journeys of Olga, Monica and Jane: their trajectories all included a successful transition from being oppressed by pain and depressive symptoms toward reclaiming agency from the pain and regaining some normalcy and control over their life. The trajectories are also consistently positive, so that there are visible improvements when comparing their drawings from T1 to T2 and from T2 to T3. The narratives reflect these improvements, also illuminating the idiosyncratic events that characterized the change within each of their lives.

At T1 all women in this trajectory group represented their pain as an oppressor having an overwhelming presence in their drawing: Monica was exposed to the whims of an evil being engulfed in a fog of pain; Olga was so oppressed that she was faceless, with only pain on her body and a heavy cloud over her mind; and Jane was a small figure crushed by a one-ton weight. Each of these drawings combines different pain metaphors of the types reported in literature. Monica's aggressive monster in a dense scattering of pain symbols includes metaphors of pain as an embodied attacker and as having physical properties, while Jane's resisting figure with its overhanging one-ton weight represents pain as an external, threatening, trapping and crushing entity (10, 11, 20, 21). By representing pain as an aggressive external agentic being, these drawings convey the disempowering psychological distress caused by pain and the sense of helplessness that Monica and Jane were experiencing at the time.

Olga's stick figure drawing, although apparently simpler and more literal in its description of pain symptoms, is more graphic than the others. It associates each type of pain to a specific implement attacking the body (pins and hammers) and illustrates pain in the lower back through the common experience of toothache (20), eliciting an almost physical recoiling response in a careful viewer. Olga's stick figure also has a blank face overhung by a dense cloud, suggesting a depersonalizing pain accompanied by depressive symptoms. Quantitative evidence has linked the use of particular pain metaphors to different types of distress, with pressure and weight pain metaphors associated to higher levels of depression and stress, and metaphors of physical damage caused by sharp objects linked to higher pain interference (22).

Each in its own way, the first pain drawings of women in this trajectory communicate a sense of being overwhelmed by an unbearable physical and psychological pain. This impact is complemented and further explained by the initial Self drawings, where Monica appeared suffocated by illness, duties and responsibilities and unable to care for herself, Olga had a desolate face and felt distant from family, and Jane drew herself as crawling on a steep dark path. The accompanying narratives spoke of depression, social isolation and hopelessness. Both Monica and Jane had a history of depression, while Olga was displaying depressive symptoms at the interview. During their first interview, both Olga and Jane questioned whether their current life was worth living. Instead, Monica was desperately resisting her pain trying to save her Self from it. The Self drawings thus illustrate the known correlations between CP and depression (23) and psychological distress more widely (24), which underly many CP treatment models (5). These Self drawings are also unique in that, to our knowledge, no other CP study has invited its participants to draw themselves to probe the existential impacts of having CP, although drawings of Self have been used to investigate other conditions, such as spinal-cord injury and dementia (25, 26). Together, the T1 pain and Self drawings illustrate how, at the start of the study, participants in this trajectory group were profoundly oppressed by their pain and afflicted by extremely low mood.

The PMP was a breakthrough moment for all participants and by T2 each of them had embarked on a new life course: Monica had started therapy to tackle her history of trauma and mental health difficulties; Olga was successfully weaning herself off opioids; and Jane had left an oppressive relationship, started to work again and moved into a new home. Accordingly, Monica's and Jane's Self drawings at Time 2 were very different from their earlier ones, with Jane's dream-like house and Monica's hornet's nest respectively representing a new ecstatic happiness and a new acceptance that life could be chaotic without this being a sign of deteriorating mental health. In contrast, Olga's second Self drawing was more subdued, but also relaxed for its concern with everyday family life.

At the same time, in the pain drawings, pain became less prominent: it was relegated to fewer body parts for Olga and to one leg for Jane, while Monica's external agentic pain was replaced by pain management strategies. A visible change had occurred for all three women and their second interviews shared a sense of relief from having broken free from previous constraints (i.e., Monica's fear of losing herself, Olga's obnubilation by morphine and Jane's toxic relationship). Each woman was expressing a new understanding of herself and her condition, starting to feel like herself again and daring to hope for the future.

The relatively short period between T1 and T2 was a turning point for these participants, with the PMP acting as a trigger leading to “changing perceptions, identities and understandings, and opening up the possibility of an alternative pathway for the future” (27). Indeed, the PMP and CCPS stimulated all three women to reconsider their life, break old patterns of behavior and challenge their own status quo. The cathartic quality of Monica's and Jane's Self drawings, particularly in comparison to their earlier drawings, conveys this sense of radical transformation. Olga's Self drawing is more proportionate and serene compared to her previous one, and illustrates her engagement with normal life.

Five months later, at T3, the progression toward normality appeared steady for all women. Pain drawings changed to give the pain even less space: Monica's pain was a careful balance of self-management strategies counterweighing life stressors; Olga's was a larger version of her smiling, with limited soft pink pain, in contrast to her previous bright red pain; Jane relegated her pain to the background, representing daily chores on the front of the J which represented her. The pain had not disappeared, but it was now manageable, and the drawings showed how: Monica's carefully balanced scale, Olga's light colors and smile and Jane's background pain with a front including “*pain relief* ” and “*rest*”. The accompanying Self drawings were images of serene normal lives, with a predominance of green, which appeared in Olga's words, Monica's plain and Jane's path. The form that normality took for each of them was different: for Olga it was making plans to tackle her ongoing obesity, for Monica it was clearing up her life and taking care of her “*Little Me*” and for Jane it was a life scattered with surmountable obstacles.

In response to their changing pain drawings, all three women talked of having taken back control from their pain, expressing a sense of agency and empowerment which had previously not been present in their narratives. They talked of finally feeling in control of their lives and able to take decisions as they had previously felt unable to do. Feeling empowered is considered an enabler for pain self management (28).

Another aspect that emerged for all women was the ability to focus on their own needs. It can be detected in Olga's “*I know to ask for help*” statement in her Self drawing and in her decision to finally lose weight, in Jane's inclusion of “*Rest*” and “*Pain relief* ” in her pain drawing and in Monica's listing “*Rest*,” “*Compassion*,” and “*Be kind to myself* ” among her self-management strategies in her pain drawing. All women were prioritizing their own wellbeing in a way that they had not done before. A useful construct to understand this change is self-compassion, which is receiving growing attention in CP, and includes being understanding toward oneself rather than self-critical (29). It is considered an adaptive process that can help reduce the impact of CP on the life of sufferers. Higher self-compassion is associated with lower depression, disability and pain-related fear, and greater pain acceptance, successful engagement in valued activities and use of pain coping strategies (30). Higher self-compassion has also been found to predict lower depressive symptoms at 6 and 12 months (31).

If we review Monica's trajectory through a self-compassion lens, we see how at T1 she had been entirely focused on looking after others and finding it difficult to look after herself. In her first Self drawing she appeared in a small green basket overwhelmed by balloons representing her burdens (her duties, her family, her illness). At T3, she drew herself as two Russian-doll figures on a serene green path, with the inner doll representing her “*Little Me*” whose happiness the outer one was protecting. Her focus had changed substantially: being nice to herself had become a priority and she was aware that it was up to her to prioritize her needs over the needs of others. Jane's decision to leave her partner was also dictated by a recognition that her own needs should take priority, as Olga's desire to lose weight stemmed from her wanting to finally take care of her body. We see here how self-compassion and regaining control are interlinked: being in control enables self-compassionate behaviors and the sense of self-compassion is empowering.

The PMP seemed to have been an empowering experience for these women: it stimulated in them a desire to take responsibility for their own wellbeing and gave them the tools to rethink their life with pain and take action toward managing it, each in her own way. The ACT psychological flexibility model (32) offers a useful framework to understand what may have happened. The model advocates for a shift from experiential avoidance to experiential acceptance, which, for CP, means acceptance of the physical and psychological experiences of pain. The reduced space occupied by the pain in their drawings over time and these participants' return to some “normality” is a testament to their ability to accept and incorporate pain and its consequences into their lives. Becoming psychologically flexible also entails a shift from being attached to one's concept of oneself and unable to act, to a more flexible view of oneself where, driven by actions in tune with one's personal values, a more vital life can be lived. The ability of participants in this group to challenge their view of themselves, reassess their lives and engage with what was important to them by taking concrete actions suggests a good degree of psychological flexibility. In a recent IPA study investigating perspectives on acceptance following an ACT-based PMP, participants spoke of acceptance as a journey encompassing enhanced self-efficacy, altered and flexible self-identity and openness to change (33), three experiences that resonate with what happened to participants in this trajectory group. Biguet, Nilsson Wikmar (34) talk of “acceptance as personal empowerment” (p. 1261), which they identify as the best possible outcome for CP sufferers, while Toye, Belton (35) see empowerment as a necessary step in the CP sufferer's journey toward healing. The sense of control over their pain developed by Olga, Monica and Jane over time, their growing sense of empowerment and the practical and psychological changes they underwent during the study suggest they were on a steady march on their path to healing.

The three women in this trajectory group started from a very low point and by the end were feeling much better in themselves and had made important changes to their lives. Meta-analytic evidence on PMP efficacy (36) points to group-based PMPs having significant effects on disability/function, pain intensity, psychological health, general health and quality of life, mainly with medium effect sizes. The women themselves attributed a role to the CCPS and PMP in their change processes. For Jane, understanding the multidimensionality of pain had been key: she compared pain to a large “*wall*” that needed to be dismantled “*brick by brick*”. The CCPS had helped Jane reconceptualize CP into its different constituent components, which made changing the aspects of her life that needed to change more feasible. For Olga the CCPS helping her to come off morphine was a “*pivotal point*” and the PMP had shown her that she was “*not alone in this sort of spiral of painkillers and pain and feeling miserable and isolated*,” stimulating reflections on her future. For Monica, the PMP and the CCPS counseling had “*set the ball rolling*” in terms of her understanding of herself and her condition. Understanding the biopsychosocial nature of pain, being with similar others, and understanding oneself better are outcomes from PMP programs considered self-management enablers (28).

Causal conclusions are always problematic in research, particularly when phenomena occur in a complex environment, where multiple events, motives and meanings interact, yet a careful longitudinal analysis can reveal interesting details of how processes unfold (27). At least partly in response to the stimuli they received from the CCPS and the PMP, the women in this trajectory group had taken life-changing decisions which had made them feel better, reclaimed agency from their pain, expressed new feelings of self-compassion, gained a sense of control over their lives, and were able, by the end of the study, to live an almost ‘normal' and serene life that would have been unthinkable at the start of the study.

The trajectories of all three women in this study confirm that CP is more than a biopsychosocial experience, it is a profoundly idiosyncratic one, where different aspects of the lifeworld of participants come into play, in a “relational and emergent process of sense-making through the lived body that is inseparable from the world that we shape and that shapes us” (37). Thanks to the level of detail inherent in a LIPA design, and the use of pain and Self drawings, the idiosyncratic sense-making of participants was contextualized within each person's particular set of circumstances.

From a methodological standpoint, this study confirms the value of using participant drawings alongside IPA interviews to obtain a more nuanced account of experience. Here, drawings of pain were accompanied by drawings of Self. The order in which drawings were produced may have influenced the imagery chosen by participants. It would be interesting in a future study to ask participants to draw themselves before drawing their pain.

This study offers readers an opportunity to immerse themselves in the experience of CP of three women who embraced PMP participation as a life-changing opportunity. The results offer a nuanced account of the interaction between the input participants received from the CP service and their responses, actions and sensemaking around CP and their life more widely. The study also offers an example of how CP constructs such as acceptance and self-compassion can be situated and illustrated in practice. Finally, although this was not the aim of this study, its results exemplify how IPA might be used longitudinally to understand the outcome of an intervention.

## Data availability statement

The paper is self-sufficient we do not feel the need to make further data available. The raw data, although anonymized, includes sensitive material which is not suitable for general distribution.

## Ethics statement

The study was reviewed and approved by London Stanmore Research Ethics Committee of the NHS Health Research Authority REC (15/LO/1872). The patients/participants provided their written informed consent to participate in this study.

## Author contributions

IEN gathered the data and took the lead on analysis and writing. JAS and JAK contributed to the research at appropriate points. All authors contributed to design the study.

## Funding

IEN received funding for the research by Birkbeck University of London through an Anniversary Scholarship.

## Conflict of interest

The authors declare that the research was conducted in the absence of any commercial or financial relationships that could be construed as a potential conflict of interest.

## Publisher's note

All claims expressed in this article are solely those of the authors and do not necessarily represent those of their affiliated organizations, or those of the publisher, the editors and the reviewers. Any product that may be evaluated in this article, or claim that may be made by its manufacturer, is not guaranteed or endorsed by the publisher.

## Footnotes

^1^A case study containing a more detailed analysis of Jane's pain drawings and with limited overlap with the current paper has already been published in: Nizza IE, Smith JA, Kirkham JA. ‘Put the illness in a box': A longitudinal interpretative phenomenological analysis of changes in a sufferer's pictorial representations of pain following participation in a pain management programme. *British Journal of Pain*. 2018;12(3):163-70.
